# Effect of staining solutions on the surface properties and color stability of conventional and CAD/CAM interim restorative materials

**DOI:** 10.4317/jced.64170

**Published:** 2026-09-15

**Authors:** Otavio Marino dos Santos-Neto, Marisa Alves Lúcio Ferreira, João Paulo do Vale-Souza, Ricardo Alexandre Zavanelli, José Vitor Quinelli Mazaro, Adriana Cristina Zavanelli

**Affiliations:** 1 Federal University of Santa Catarina, Florianópolis, SC, Brazil; 2 São Paulo State University (UNESP), School of Dentistry, Araçatuba, SP, Brazil; 3 Federal University of Goiás, Goiânia, GO, Brazil

**Keywords:** Additive manufacturing, CAD/CAM, interim restorations, color stability, surface properties.

## Abstract

**Background:**

The long-term esthetic and physical performance of interim restorations depends on the material’s resistance to the oral environment; however, the impact of common beverages on newer CAD/CAM and 3D-printed resins remains unclear. The purpose of this in vitro study was to evaluate the surface roughness (ΔRa), microhardness (KHN), and color stability (ΔE00) of 5 interim restorative materials after immersion in different staining solutions.

**Materials and Methods:**

Specimens (N=200, n=10 per group) were fabricated from autopolymerizing acrylic, heat-polymerized acrylic, bis-acryl, milled CAD/CAM (PMMA), and 3D-printed resins. After 30 days of distilled water storage, baseline measurements were recorded. Specimens were then immersed in coffee, a cola-based beverage, red wine, or artificial saliva for 14 days, with daily solution renewal. Data were analyzed using ANOVA, Kruskal-Wallis, paired t-tests, and Wilcoxon tests as appropriate (α=.05).

**Results:**

Red wine resulted in significant differences in surface roughness (P=.018), increasing ΔRa for the 3D-printed resin (0.93 ± 1.25) while decreasing it for milled PMMA (-1.01 ± 1.20). Microhardness significantly decreased for most material-solution combinations after 14 days (P<.05). Coffee and red wine caused the greatest color changes, with the bis-acryl resin exhibiting ΔE00 values up to 4.05 ± 2.09, exceeding the clinical acceptability threshold (1.8). CAD/CAM-based materials generally demonstrated greater color stability than conventional materials.

**Conclusions:**

Milled CAD/CAM interim resins demonstrated superior surface and color stability compared with conventional and 3D-printed materials. Red wine negatively impacted the surface integrity of 3D-printed resins, while bis-acryl materials were most susceptible to clinically unacceptable staining.

## Introduction

Interim restorations are essential components of restorative treatment, serving to protect pulp tissue, provide periodontal support, and maintain occlusal stability while the definitive prosthesis is being fabricated [1]. High-performance polymers, including milled and 3D-printed CAD/CAM resins, have been introduced to overcome the mechanical and aesthetic limitations of conventional autopolymerizing and heat-polymerized acrylic resins [2,3].

However, the prolonged exposure of these materials to the oral environment makes them susceptible to degradation. Surface roughness (Ra) and microhardness are critical properties, as increased roughness facilitates biofilm accumulation and staining, while reduced hardness may compromise the wear resistance of the restoration [4,5]. Furthermore, color stability is a major concern, particularly for patients with high consumption of staining beverages such as coffee and red wine [6].

The staining process is complex and involves both intrinsic factors, related to the polymer matrix and filler content, and extrinsic factors, such as the chemical composition of the staining solutions [7]. While milled CAD/CAM resins are polymerized under high pressure and temperature, resulting in high density and low porosity, 3D-printed resins rely on layer-by-layer photopolymerization, which may leave residual monomers and influence their long-term stability [8,9].

Although several studies have evaluated the properties of interim restorative materials [2,4,8-11], differences in materials, evaluated properties, and staining protocols may still limit direct comparisons among studies. To address this gap, the novelty of the present study lies in the simultaneous comparison of conventional, milled CAD/CAM, and 3D-printed interim materials under standardized immersion protocols, evaluating surface roughness, microhardness, and color stability together.

The purpose of this *in vitro* study was to evaluate the effect of staining solutions on the surface roughness, microhardness, and color stability of conventional and CAD/CAM interim restorative materials. The null hypothesis was that no significant differences would be found among the materials regarding their surface properties and color stability, regardless of the staining solution used.

## Materials and Methods 

The interim restorative materials evaluated in this study, including brand names, manufacturers, and chemical compositions, are summarized in Table 1. Disk-shaped specimens (14.8 ± 0.2 mm in diameter and 2.7 ± 0.2 mm in thickness) were fabricated from 5 materials in accordance with the ISO 20795 standard. Autopolymerizing acrylic resin, heat-polymerized acrylic resin, and bis-acryl resin specimens were fabricated using a stainless-steel mold previously lubricated with a water-based lubricant. The acrylic resins were manipulated according to the manufacturers’ recommendations, inserted into the mold, and submitted to hydraulic pressing (1000 kgf). The heat-polymerized acrylic resin was processed in a water bath at 100 °C for 40 minutes, whereas the autopolymerizing and bis-acryl resins were allowed to polymerize at room temperature. The milled CAD/CAM PMMA specimens were designed using modeling software (ArtCAM; Autodesk, San Rafael, Calif) and milled from PMMA blocks. The 3D-printed specimens were fabricated from a photopolymerizable resin using additive manufacturing, washed in isopropyl alcohol, and post-polymerized in a dedicated curing unit.

All specimens were initially finished with a tungsten carbide rotary instrument and polished under constant water cooling using sequential silicon carbide abrasive papers (600- and 1200-grit) in a manual polishing machine. Final dimensions were verified with a digital caliper. To reduce residual monomer content specimens were stored in distilled water for 30 days before baseline testing. The overall experimental workflow is illustrated in Figure 1. Subsequently, the specimens were distributed into 4 immersion solutions: A cola-based beverage, coffee (2 g of powder per 50 mL of hot distilled water), red wine, and artificial saliva (control). The commercial names and compositions of the solutions are detailed in Table 2. Each specimen was individually immersed in 4 mL of the respective solution using separate disposable syringes to prevent cross-contamination. The immersion period was 14 days, with daily renewal of the solutions.

Surface roughness was measured using a surface profilometer (SJ-400; Shimadzu Corp, Tokyo, Japan) with a 0.8-mm cutoff, a measurement length of 1.5 mm, and a speed of 0.8 mm/s. Three readings were obtained per specimen in different directions, and the mean variation (ΔRa) was calculated. Knoop microhardness was evaluated using a microhardness tester (HMV-G; Shimadzu Corp, Tokyo, Japan) with a 50-g load applied for 10 seconds. Three indentations were made per specimen to determine the Knoop Hardness Number (KHN). Color change was evaluated using a spectrophotometer (UV-2450; Shimadzu Corp, Tokyo, Japan) in a metamerism box under simulated daylight conditions with white and black backgrounds. Color parameters were recorded using the CIELab system, and color differences (ΔE_00_) were calculated using the CIEDE2000 formula. The clinical acceptability threshold for color difference was defined as ΔE_00_=1.8, according to Paravina *et al*. [12].

Statistical analysis was performed using software (IBM SPSS Statistics, v25.0; IBM Corp, Armonk, NY). Data normality was verified with the Shapiro-Wilk test. For comparisons among materials within each immersion solution, 1-way ANOVA followed by the Tukey post hoc test was used for normally distributed data. The Kruskal-Wallis test followed by the Dunn post hoc test with Bonferroni correction was applied for non-normally distributed data. For microhardness, paired comparisons between baseline and 14-day measurements were performed using paired t-tests or Wilcoxon signed-rank tests according to data distribution (α=.05).

## Results

Surface roughness variation (ΔRa) after 14 days of immersion is shown in Table 3. 

No statistically significant differences in surface roughness were observed among the materials after immersion in coffee, the cola beverage, and artificial saliva (*P*>.05). However, immersion in red wine resulted in significant differences among the materials (*P*=.018). Under this condition, the 3D-printed CAD/CAM resin exhibited the highest increase in surface roughness, whereas the milled CAD/CAM PMMA resin demonstrated a reduction in surface roughness.

Knoop microhardness (KHN) values at baseline and after 14 days of immersion are detailed in Table 4 and Figure 2. Paired comparisons revealed a statistically significant reduction in microhardness for most material-solution combinations after 14 days of immersion (*P*<.05). However, no significant changes were observed for the autopolymerizing acrylic resin after immersion in coffee, cola, and artificial saliva, or for the 3D-printed CAD/CAM resin after immersion in cola (*P*>.05). Furthermore, significant differences among the materials were observed within each solution group (*P*≤.004). The 3D-printed CAD/CAM resin consistently exhibited the lowest microhardness values both at baseline and after immersion, while the remaining materials showed intermediate behavior with slight numerical variations.

Color change (ΔE_00_) values after 14 days of immersion are presented in Table 5 and Figure 3. Significant differences among the materials were observed across all immersion solutions (*P*<.05). Overall, artificial saliva resulted in the lowest color variation, whereas coffee and red wine resulted in the highest ΔE_00_ values. The bis-acryl resin showed the highest susceptibility to staining, particularly in red wine (4.05 ± 2.09). Conversely, the milled CAD/CAM PMMA resin exhibited the highest color stability, maintaining the lowest ΔE_00_ values across all tested conditions.

## Discussion

The aim of this study was to evaluate the surface roughness, microhardness, and color stability of different provisional restorative materials after immersion in staining solutions over a 14-day period. Based on the results obtained, the null hypothesis was rejected, as the different materials exhibited distinct behaviors after the immersion period. These findings indicate that both the type of material and the immersion solution significantly influenced the evaluated properties, which is consistent with previous studies reporting material-dependent physicochemical and optical changes in provisional and CAD/CAM resins exposed to staining agents [13-16]. Furthermore, by simultaneously evaluating surface roughness, microhardness, and color stability under standardized conditions, this study provides a comprehensive profile of material degradation. This integrated approach highlights the differential behavior of conventional, milled, and 3D-printed materials when exposed to the same chemical challenges, offering a broader perspective on their overall durability and guiding material selection more effectively than isolated assessments.

Regarding surface roughness, only red wine resulted in statistically significant differences among the tested materials, whereas coffee, cola-based beverage, and artificial saliva did not result in significant intergroup differences. After immersion in red wine, the 3D-printed CAD/CAM resin exhibited the highest increase in surface roughness, while the milled CAD/CAM resin (PMMA) showed greater surface stability, with reduced ΔRa values. This behavior reinforces the influence of material composition and manufacturing method on surface characteristics [17,18].

This pattern may be explained by the chemical interaction between the immersion medium and the polymeric matrix. Acidic and alcoholic solutions can promote liquid sorption, matrix plasticization, and leaching of organic components-mechanisms commonly described for resin-based materials exposed to challenging chemical environments [19,20].

Differences related to fabrication methods may also explain these findings. Milled CAD/CAM materials are industrially polymerized, resulting in a more homogeneous structure and a higher degree of conversion. In contrast, 3D-printed resins present a layer-by-layer architecture, whose mechanical and surface properties depend on printing parameters and post-curing protocols [8,9]. In addition, recent studies have shown that surface treatments such as polishing, glazing, and coating can significantly influence roughness and staining susceptibility, particularly in additively manufactured materials [21,22].

These structural differences have important biological implications, as increased surface roughness is associated with higher microbial adhesion and biofilm formation [1,23]. Although the polishing procedures adopted in this study were standardized and laboratory-based, the observed changes may have clinical relevance, as surface condition plays a key role in both biological and esthetic performance [24]. Microhardness analysis demonstrated a significant reduction in this property after 14 days of immersion for the majority of the evaluated materials. However, the autopolymerizing acrylic resin and the 3D-printed CAD/CAM resin exhibited stable microhardness values in specific immersion conditions, indicating variable susceptibility to hydrolytic degradation. This finding is consistent with previous studies showing that provisional materials undergo mechanical degradation after exposure to aging conditions [25,26].

The 3D-printed CAD/CAM resin consistently exhibited lower microhardness values compared with the other materials, suggesting greater susceptibility to degradation. In contrast, heat-polymerized acrylic resin and milled CAD/CAM PMMA generally maintained higher microhardness values, which may be attributed to their higher degree of polymerization and more homogeneous polymer network [27-29]. These findings are in agreement with previous investigations reporting improved mechanical performance of CAD/CAM milled materials and variable behavior of additively manufactured resins depending on processing conditions [2,26,30].

The reduction in microhardness can be attributed to water sorption and hydrolytic degradation of the polymer matrix. Water absorption promotes expansion between polymer chains, reducing intermolecular forces and leading to surface softening [5,31].

Regarding color stability, all immersion solutions resulted in measurable chromatic alterations, with statistically significant differences among materials under all conditions. Color changes were more pronounced in coffee and red wine, whereas artificial saliva showed minimal color change. This behavior is consistent with the known staining potential of these substances [4,6].

The bis-acryl resin exhibited greater color change across most conditions, which is clinically relevant given its frequent use in esthetic provisional restorations. In contrast, the milled CAD/CAM resin (PMMA) showed lower ΔE_00_ values, indicating greater color stability. These findings are in agreement with previous studies demonstrating superior optical stability of industrially polymerized materials compared with conventional and additively manufactured resins [15,16,29]. The 3D-printed CAD/CAM resin showed intermediate performance, which may be influenced by its microstructural characteristics and the presence of residual monomers or variations in post-curing protocols [2,8,22]. 

Beyond statistical significance, the color changes observed in this study have relevant clinical implications when evaluated against established visual thresholds [12,30]. While specimens immersed in artificial saliva showed minimal color variation, exposure to coffee and red wine resulted in higher ΔE_00_ values. Some materials, particularly the bis-acryl resin, exhibited changes exceeding the clinical acceptability threshold (ΔE_00_>1.8), indicating clinically unacceptable discoloration. In contrast, the milled CAD/CAM resin consistently demonstrated lower color variation, often remaining within clinically acceptable limits, corroborating previous findings that reported superior color stability for CAD/CAM provisional materials [13,29].

Although the 14-day immersion period does not fully reproduce long-term clinical conditions, the results provide important insights into the early behavior of provisional restorative materials. In clinical practice, these materials are often used for short periods, especially in esthetic zones, and early degradation may already compromise their performance [2,4].

Some limitations of this study should be considered. As an *in vitro* investigation, it does not fully replicate the complexity of the oral environment, including thermal cycling, mechanical brushing, and masticatory forces [31]. Additionally, only one polishing protocol and a relatively short immersion period were evaluated. Future studies should incorporate longer aging periods and combined mechanical and chemical challenges to better simulate clinical conditions.

## Conclusions

Within the limitations of this *in vitro* study, the following conclusions were drawn: The milled CAD/CAM (PMMA) resin showed greater surface and color stability under the tested conditions and may be considered a favorable option for interim restorations requiring enhanced esthetic stability. Microhardness was affected by immersion, with most materials showing a reduction after 14 days. The 3D-printed CAD/CAM resin consistently exhibited the lowest microhardness values among the materials evaluated. The bis-acryl resin was the most susceptible to color change, particularly after immersion in coffee and red wine, whereas CAD/CAM-based materials generally demonstrated greater color stability. 

## Figures and Tables

**Table 1:** Table 1: Interim restorative materials evaluated in the study, including commercial name, manufacturer, and basic composition.

Material Type	Commercial Name	Manufacturer	Basic Composition
Self-curing acrylic resin	Clássico - Shade 61	Clássico	Liquid: Methyl methacrylate monomer, Topanol. Powder: Methyl ethyl methacrylate copolymer, dibutyl phthalate (DBP), pigments, peroxide.
Heat-polymerized acrylic resin (bivalent)	Evocor - Shade 1A	Evoden	Powder: PMMA, benzoyl peroxide, biocompatible pigments. Liquid: Methyl methacrylate, EDMA, inhibitor, fluorescent agent.
Bis-acryl resin	Structur - Shade A1	VOCO	Dimethacrylates, silicic acid, initiators, diacrylate, stabilizers, synthetic resins, pigments, dyes, and strontium glass powder.
Milled CAD/CAM resin (PMMA)	Vipiblock - Shade A1	VIPI	PMMA, polymerized ethylene glycol dimethacrylate (EDMA), organically modified ceramic (OMC), fluorescent agent, DLC.
3D-printed CAD/CAM resin	Prizma 3D Bio Prov - Shade A1	Makertech Lab / Maquira	Oligomers, monomers, photoinitiators, stabilizers, and pigments.

**Table 2:** Table 2: Immersion solutions used in the study, including commercial name, manufacturer, and composition.

**Solution**	**Commercial name**	**Manufacturer**	**Composition**
Cola-based beverage	Coca-Cola	Coca-Cola Indústrias Ltda	Carbonated water, sugar, cola nut extract, caffeine, caramel color IV, phosphoric acid, natural flavor
Coffee	Melitta Instant Coffee	Melitta do Brasil Indústria e Comércio Ltda	Soluble coffee
Red wine	Casillero del Diablo	Viña Concha y Toro S.A.	Wine grapes (Cabernet Sauvignon), preservative (sulfur dioxide, INS220)
Artificial saliva	-	Apothimed Compounding pharmacy	1.5 mmol/L Ca(NO_3_)_2_·4H_2_O, 0.9 mmol/L NaH_2_PO_4_·2H_2_O, 150 mmol/L KCl, 0.1 mol/L Tris-buffered solution, 0.03 ppm F, pH 7.0

**Table 3:** Table 3: Surface roughness variation (ΔRa, µm) of the evaluated resins after 14 days of exposure to the evaluated solutions.

**Solution**	**Bis-acryl resin**	**Heat-polymerized acrylic resin**	**3D-printed CAD/CAM resin**	**Milled CAD/CAM resin (PMMA)**	**Autopolymerizing acrylic resin**	*p value*
Coffee	0.11 ± 0.22	0.05 ± 0.89	0.78 ± 2.01	-0.36 ± 1.16	0.05 ± 0.88	0.435
Cola	0.11 ± 0.28	0.13 ± 0.72	0.52 ± 1.35	-0.52 ± 0.96	0.22 ± 0.68	0.237
Red wine	-0.04 ± 0.09^B^	0.18 ± 0.52^AB^	0.93 ± 1.25^A^	-1.01 ± 1.20^ B^	0.07 ± 0.98^AB^	0.018*
Saliva	0.03 ± 0.15	0.81 ± 2.02	0.16 ± 0.53	-0.32 ± 1.15	0.17 ± 1.19	0.348

ΔRa = surface roughness after 14 days - baseline surface roughness. Values are expressed as mean ± standard deviation. Different superscript letters indicate statistically significant differences among materials within the same solution (one-way ANOVA or Kruskal-Wallis test, according to data distribution, followed by appropriate post-hoc tests, p<0.05). *Statistically significant differences among materials within each solution (p<0.05).

**Table 4:** Table 4: Knoop microhardness (KHN) of the evaluated resins according to immersion solution and time.

**Solution**	**Time**	**Bis-acryl resin**	**Heat-polymerized acrylic resin**	**3D-printed CAD/CAM resin**	**Milled CAD/CAM resin (PMMA)**	**Autopolymerizing acrylic resin**	*p value* **†**
Coffee	Initial	19.13 ± 4.23ᴬᴮᵃ	21.83 ± 4.46ᴬᵃ	13.20 ± 0.74^Ca^	17.47 ± 3.22ᴮᵃ	12.67 ± 1.21^Ca^	<0.001*
	14 days	15.30 ± 2.00ᴬᴮᵇ	16.40 ± 4.80ᴬᵇ	11.70 ± 0.80^Cb^	14.80 ± 1.60ᴮᵇ	12.50 ± 0.90^Ca^	<0.001*
Cola	Initial	21.30 ± 3.76ᴬᵃ	20.00 ± 1.13ᴬᵃ	13.33 ± 1.07^Ca^	18.17 ± 2.00ᴮᵃ	12.57 ± 0.65^Ca^	<0.001*
	14 days	14.90 ± 1.74ᴬᵇ	15.40 ± 2.20ᴬᵇ	13.10 ± 1.20^Ba^	15.60 ± 1.30ᴬᵇ	12.70 ± 0.80^Ba^	0.002*
Red wine	Initial	20.93 ± 3.42ᴬᵃ	21.67 ± 2.15ᴬᵃ	15.00 ± 3.15^Ca^	18.40 ± 1.91^Ba^	13.23 ± 1.19^Ca^	<0.001*
	14 days	14.20 ± 1.60ᴮᵇ	15.60 ± 2.40ᴬᵇ	11.10 ± 0.60^Cb^	13.60 ± 0.80ᴮᵇ	12.10 ± 1.20^Cb^	<0.001*
Saliva	Initial	19.43 ± 2.90ᴬᴮᵃ	20.97 ± 2.58ᴬᵃ	13.50 ± 0.81^C^ᵃ	18.47 ± 1.91ᴮᵃ	13.33 ± 1.54^C^ᵃ	<0.001*
	14 days	16.00 ± 2.50ᴬᵇ	15.70 ± 2.30ᴬᵇ	11.80 ± 0.70^C^ᵇ	15.80 ± 1.90ᴬᵇ	13.80 ± 1.70ᴮ^a^	0.004*

Values are expressed as mean ± standard deviation. Uppercase letters indicate statistically significant differences among materials within the same solution and time point (Kruskal-Wallis/Dunn, p<0.05). Lowercase letters indicate statistically significant differences between initial and 14-day measurements within each material (paired tests, p<0.05). †p values refer to comparisons among materials. *Statistically significant.

**Table 5:** Table 5: Color change (ΔE_00_) of the evaluated resins after 14 days of exposure to the evaluated solutions.

**Solution**	**Bis-acryl resin**	**Heat-polymerized acrylic resin**	**3D-printed CAD/CAM resin**	**Milled CAD/CAM resin (PMMA)**	**Autopolymerizing acrylic resin**	*p value*
Coffee	3.06 ± 0.90ᵃ	2.63 ± 0.17ᵃ	1.48 ± 0.44ᵇ	2.75 ± 1.94ᵃᵇ	2.58 ± 0.77ᵃ	<0.001*
Cola	2.81 ± 0.79ᵃ	1.89 ± 0.54ᵇ	1.75 ± 1.45ᵇ	1.24 ± 0.56ᶜ	1.87 ± 0.59ᵇ	<0.001*
Red wine	4.05 ± 2.09ᵃ	2.66 ± 0.43ᵃ	2.64 ± 2.04ᵃ	1.20 ± 0.36ᵇ	2.68 ± 0.92ᵃ	<0.001*
Saliva	1.76 ± 1.29ᵃ	1.54 ± 0.29ᵇ	1.06 ± 0.24ᶜ	1.07 ± 0.13ᶜ	1.37 ± 0.35ᵇ	0.0017*

Values are expressed as mean ± standard deviation. For comparisons among materials within each solution, one-way ANOVA followed by Tukey’s post-hoc test was used, except when data normality was not confirmed, in which case the Kruskal-Wallis test followed by Dunn’s post-hoc test was applied. Different superscript letters indicate statistically significant differences among materials within the same solution (p<0.05). Based on Paravina et al. [12], a clinical acceptability threshold of ΔE_00_=1.8, was adopted to evaluate the clinical relevance of the color changes.

**Figure 1:**
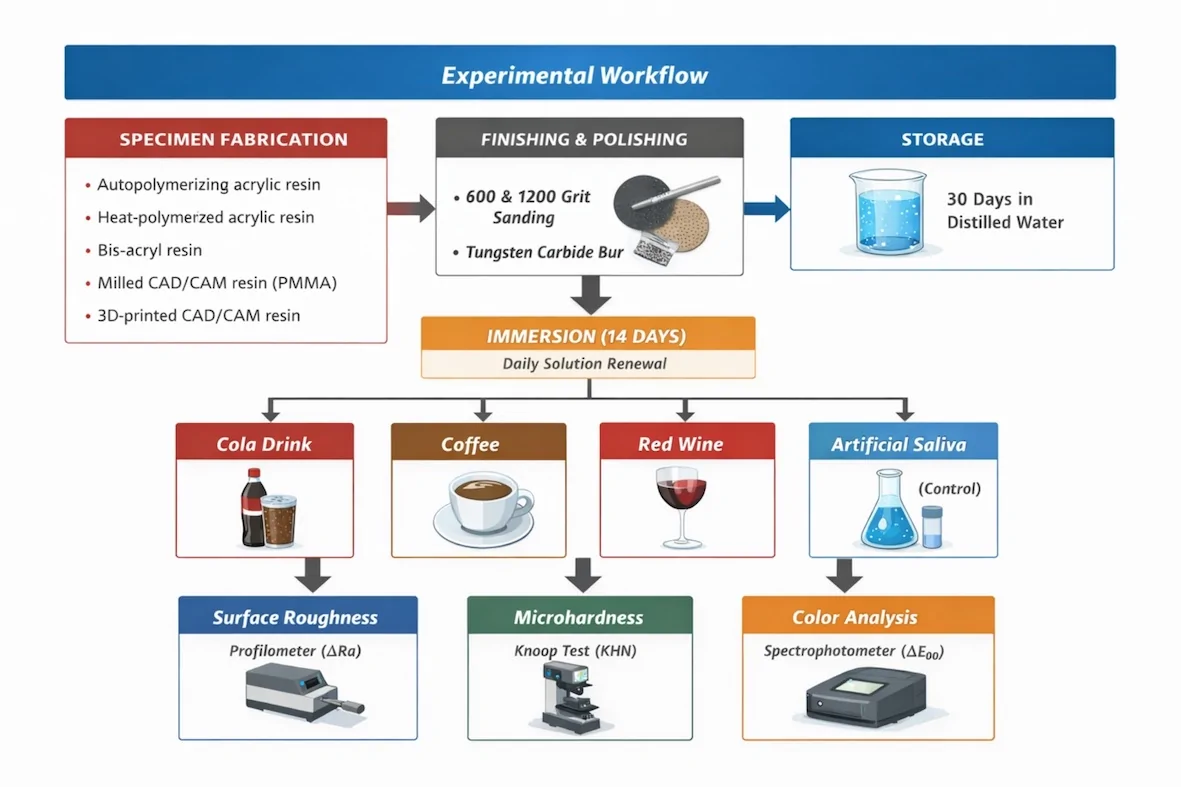
Fig. 1: Experimental workflow including specimen fabrication, standardization, immersion protocol, and measurements of surface roughness (ΔRa), microhardness (KHN), and color change (ΔE_00_).

**Figure 2:**
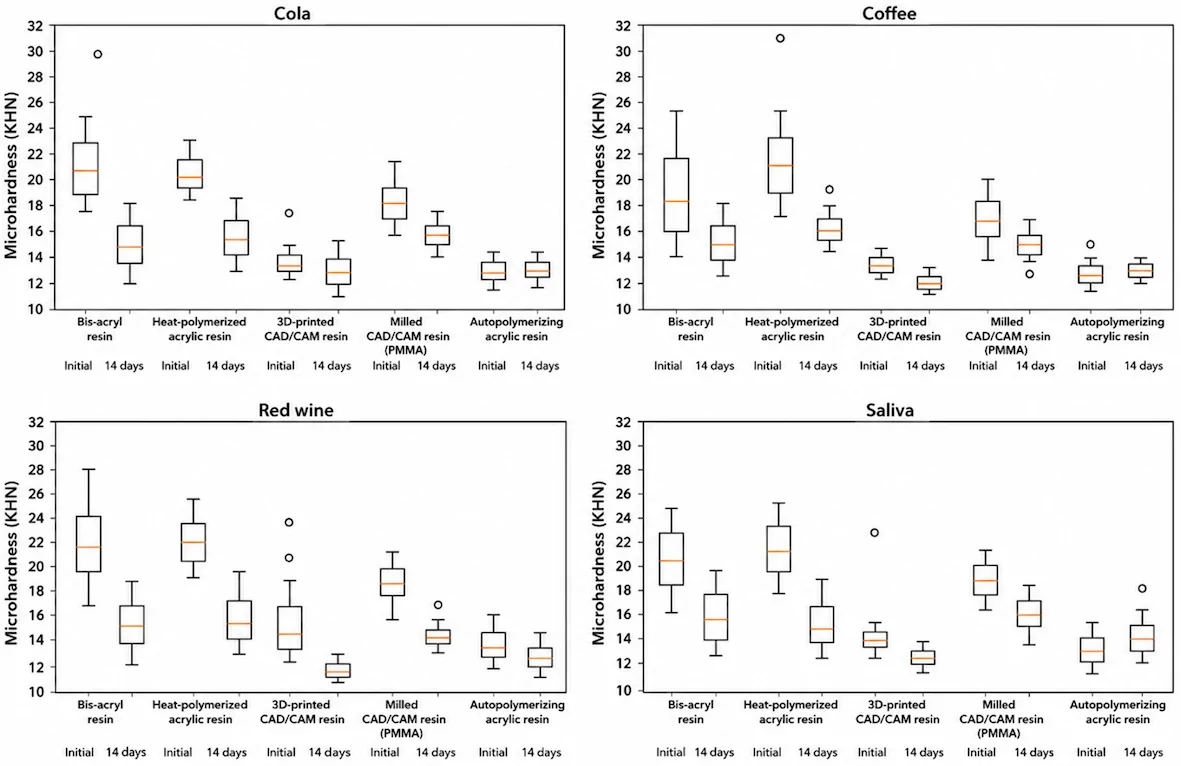
Fig. 2: Boxplots representing the distribution of microhardness values of the evaluated interim restorative materials at baseline (Initial) and after 14 days of immersion in cola, coffee, red wine, and artificial saliva.

**Figure 3:**
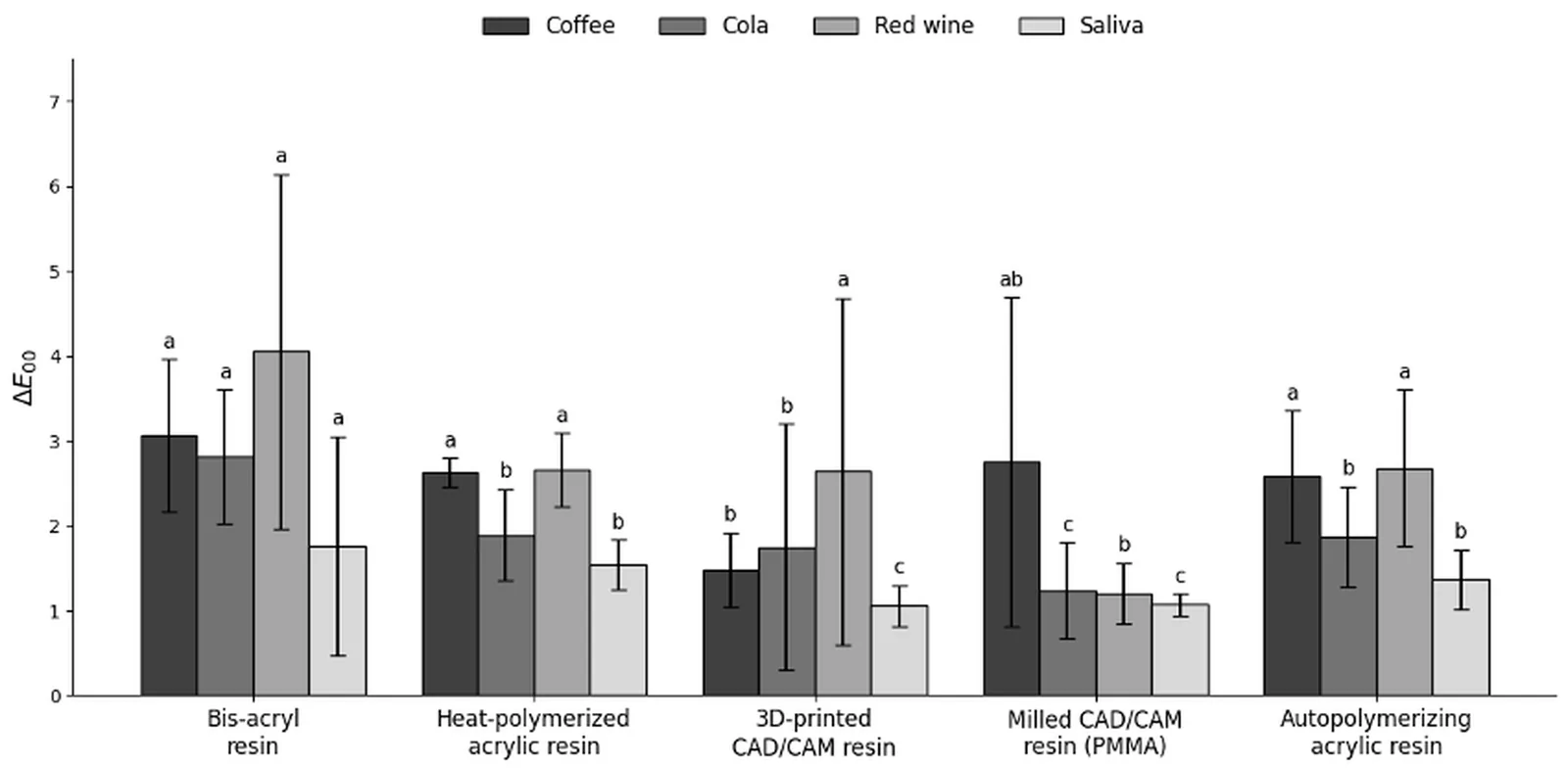
Fig. 3: Color change (ΔE_00_) after 14 days of immersion in different solutions. Values are expressed as mean ± standard deviation. Different letters indicate statistically significant differences (p<0.05).

## Data Availability

The datasets used and/or analyzed during the current study are available from the corresponding author upon reasonable request.
